# Prognostic Value of Arterial Lactate in Predicting In-Hospital Mortality in Acute Pulmonary Embolism

**DOI:** 10.3390/diagnostics16091293

**Published:** 2026-04-25

**Authors:** Hasan Veysel Keskin, Neslihan Ozcelik, Cansu Ağralı Gündoğmuş, Elvan Senturk Topaloglu, Gonul Erkan, Songul Ozyurt, Aziz Gumus

**Affiliations:** 1 Department of Pulmonology, Ahi Evren Thoracic and Cardiovascular Surgery Training and Research Hospital, Trabzon University, 61040 Trabzon, Turkey; keskinh.veysel@gmail.com; 2 Department of Pulmonology, Faculty of Medicine, Recep Tayyip Erdogan University, 53100 Rize, Turkey; neslihan.ozcelik@erdogan.edu.tr (N.O.); songul.ozyurt@erdogan.edu.tr (S.O.); aziz.gumus@erdogan.edu.tr (A.G.); 3 Public Health Services Directorate, Trabzon Provincial Directorate of Health, 61040 Trabzon, Turkey; 4 Department of Anesthesiology and Reanimation, Ahi Evren Thoracic and Cardiovascular Surgery Training and Research Hospital, Trabzon University, 61040 Trabzon, Turkey

**Keywords:** pulmonary embolism, arterial blood gas, lactate, mortality, risk stratification, PESI

## Abstract

**Background**: Early risk assessment in acute pulmonary embolism (PE) remains challenging, particularly in normotensive patients. Lactate may offer incremental prognostic value beyond conventional tools. We investigated the association between arterial lactate and in-hospital mortality in acute PE. **Methods**: In this retrospective single-center study, 327 adult patients diagnosed with acute PE by computed tomography pulmonary angiography who underwent arterial blood gas analysis within the first six hours of emergency department presentation were included. Patients were categorized according to the occurrence of in-hospital mortality, including 103 (31.5%) non-survivors and 224 (68.5%) survivors, and their demographic, clinical, laboratory, and echocardiographic characteristics were compared accordingly. **Results**: Arterial lactate levels were significantly higher in non-survivors than survivors [4.1 vs. 1.9 mmol/L; *p* < 0.001], with a stepwise increase in mortality across lactate categories (<2, 2–4, >4 mmol/L; *p* < 0.001). In normotensive patients (*n* = 211), lactate ≥2 mmol/L was associated with higher mortality compared with <2 mmol/L (35.7% vs. 8.7%; OR 5.8, 95% CI 2.7–12.5; *p* < 0.001). In multivariable logistic regression analysis performed in normotensive patients, arterial lactate level, PESI score, and the presence of cerebrovascular disease were identified as independent predictors of in-hospital mortality, whereas troponin did not retain independent significance. In normotensive patients, lactate showed better discriminative ability than troponin I (AUC 0.718 vs. 0.553). **Conclusions**: Arterial lactate levels are independently associated with in-hospital mortality in acute PE. Elevated lactate may help identify high-risk patients even in the absence of hypotension and may provide incremental prognostic value beyond existing risk stratification tools. These findings suggest the use of arterial lactate in early risk assessment.

## 1. Introduction

Venous thromboembolism (VTE), which encompasses deep vein thrombosis (DVT) and pulmonary embolism, is among the leading causes of cardiovascular mortality worldwide and constitutes a significant burden of morbidity and mortality [1]. The recent increase in PE incidence has been attributed to heightened clinical awareness, the widespread use of multidetector computed tomography pulmonary angiography leading to higher diagnostic rates, and the contribution of the COVID-19 pandemic to thromboembolic events [2]. Given the highly heterogeneous clinical course of PE, accurate early risk stratification is critical for guiding therapeutic strategies and predicting prognosis. Current guidelines define the presence of hemodynamic instability as a high-risk criterion, whereas in normotensive patients, risk stratification is based on the presence of right ventricular (RV) dysfunction and elevated cardiac biomarkers. Patients with both parameters are classified as intermediate–high-risk [3]. Thirty-day mortality rates have been reported to be approximately 30% in the high-risk group, whereas in the intermediate–high-risk group, mortality ranges between 9% and 20% [3,4,5]. Accordingly, systemic thrombolytic therapy or catheter-based interventional approaches are recommended for high-risk patients. However, although thrombolytic therapy in the intermediate–high-risk group has been shown to reduce hemodynamic deterioration and the development of shock, it is also associated with a significantly increased risk of major bleeding, particularly intracranial hemorrhage [6]. These findings suggest that patients within the intermediate–high-risk category are not a homogeneous population and underscore the need for additional prognostic markers to better identify the subgroup at higher risk of clinical deterioration [7].

Plasma lactate level is an important biochemical marker of tissue hypoperfusion and organ dysfunction in critically ill patients and is used for prognostic assessment in clinical conditions such as sepsis and trauma [8,9]. Lactate is a dynamic parameter reflecting the imbalance between tissue oxygen delivery and consumption; in addition to increased anaerobic metabolism due to hypoxia, it may also rise as a result of sympathoadrenergic activation and microcirculatory dysfunction. In acute PE, the abrupt increase in pulmonary vascular resistance results in right ventricular overload, reduced cardiac output, systemic hypoperfusion, and impaired gas exchange, which are the main pathophysiological processes leading to increased lactate production. An important point is that elevations in lactate levels may often occur before the development of overt systemic hypotension. This observation increases the potential prognostic relevance of lactate as an early indicator of subclinical circulatory failure, particularly in normotensive patients [10]. Although studies in the existing literature have demonstrated a possible association between elevated lactate levels and mortality in PE, the incremental contribution of lactate beyond current risk stratification systems, particularly in normotensive patients, needs to be clearly established, and its role in clinical practice requires further clarification.

The aim of this study was not only to evaluate the association between arterial lactate levels and in-hospital mortality in acute pulmonary embolism but also to determine whether lactate provides incremental prognostic value beyond established risk stratification tools, particularly in normotensive patients, a clinically challenging and heterogeneous subgroup.

## 2. Materials and Methods

### 2.1. Study Design

This retrospective observational study was conducted in patients diagnosed with acute PE in the emergency department of Recep Tayyip Erdoğan University Training and Research Hospital. Adult patients who were diagnosed with PE in the emergency department and whose diagnosis was confirmed by thoracic computed tomography pulmonary angiography (CTPA) were included in the study. Arterial lactate levels were obtained from arterial blood gas (ABG) samples collected within the first six hours following admission to the emergency department.

### 2.2. Participants and Data Collection

The inclusion criteria were defined as age ≥18 years, a diagnosis of acute PE confirmed by CTPA, and availability of arterial blood gas analysis performed within the first six hours after admission.

The exclusion criteria were age <18 years, absence of CTPA-confirmed PE, and lack of arterial blood gas sampling within the first six hours of presentation.

Variables with missing data were assessed prior to analysis, and patients with missing data in critical variables were excluded. All analyses were conducted using a complete-case dataset. Missing data were minimal, and no systematic pattern of missingness was observed upon exploratory analysis.

For all included patients, demographic characteristics, presenting symptoms, clinical risk factors, comorbidities, initial vital signs, and laboratory parameters were obtained from the electronic medical record system and recorded in a standardized data collection form. In addition, echocardiographic findings and lower extremity venous Doppler ultrasonography results were evaluated.

Arterial blood gas analysis was performed within the first six hours of emergency department admission based on clinical judgment, particularly in patients with more severe clinical presentation or suspected respiratory failure.

The Pulmonary Embolism Severity Index (PESI), the Simplified Pulmonary Embolism Severity Index (sPESI), and the European Society of Cardiology risk classification were calculated based on the available clinical, imaging, and laboratory data.

### 2.3. Definitions

Patients with a systolic blood pressure ≥100 mmHg were defined as normotensive. Although current ESC guidelines define hemodynamic instability as a systolic blood pressure <90 mmHg, validated risk stratification tools such as the Pulmonary Embolism Severity Index and simplified PESI use a threshold of <100 mmHg to identify higher-risk patients [3,11]. Therefore, a cutoff of 100 mmHg was used to define a clinically stable and more homogeneous normotensive population and to reduce misclassification of borderline hypotensive patients. Right ventricular dysfunction was defined as the presence of at least one of the following echocardiographic findings: right ventricular dilatation, hypokinesia, or an increased right ventricular-to-left ventricular ratio, in line with commonly used criteria in the literature [3,12].

Arterial lactate levels were measured using arterial blood gas analysis and were reported in mmol/L. High-sensitivity troponin I (hs-TnI) levels were assessed as a cardiac biomarker, and measurements were expressed in µg/L.

### 2.4. Outcome Measures

The primary endpoint of the study was defined as in-hospital mortality. Given the retrospective design and the presence of concomitant comorbidities, it was not possible to attribute mortality exclusively to pulmonary embolism in every case. Therefore, consistent with the existing literature, all-cause in-hospital mortality was used as the outcome measure.

### 2.5. Statistical Analysis

Statistical analyses were performed using IBM SPSS Statistics software (version 27, IBM Corp., Armonk, NY, USA). The distribution of continuous variables was assessed using the Kolmogorov–Smirnov test. Continuous variables not normally distributed were presented as median (minimum–maximum), while categorical variables were expressed as counts and percentages (%). Comparisons between groups were performed using the Mann–Whitney U test for continuous variables and the chi-square test or Fisher’s exact test for categorical variables, as appropriate.

Univariable and multivariable logistic regression analyses were performed to assess the association between arterial lactate levels and in-hospital mortality. Candidate variables were selected based on both clinical relevance and statistical significance in univariable analysis (*p* < 0.10). In addition, established prognostic factors and clinically important variables, including the Pulmonary Embolism Severity Index (PESI) score and relevant comorbidities, were considered for inclusion in the multivariable model regardless of their univariable significance. This approach was adopted to reduce the risk of excluding clinically meaningful confounders and to improve model robustness. Results were reported as odds ratios (OR) with 95% confidence intervals (CI). In the normotensive patient subgroup, the prognostic value of arterial lactate levels was further analyzed, and mortality rates were compared according to lactate categories (<2, 2–4, >4 mmol/L).

The predictive performance of lactate and troponin levels for mortality was assessed using ROC curve analysis, and the area under the curve (AUC) values were calculated. A two-tailed *p* value < 0.05 was considered statistically significant.

During the preparation of this manuscript, generative artificial intelligence tools were used for language editing and translation purposes. All generated content was reviewed, revised, and validated by the authors, who take full responsibility for the content of the manuscript.

## 3. Results

During the study period, a total of 379 patients with a diagnosis of acute pulmonary embolism confirmed by pulmonary angiography were evaluated. Fifty-two patients were excluded due to the absence of arterial blood gas analysis within the first six hours after emergency department admission, and the remaining 327 patients were included in the final analysis.

The median age of the study population was 75 (19–99) years; 217 patients (66.4%) were female and 110 (33.6%) were male. In-hospital mortality occurred in 103 patients (31.5%). Among normotensive patients, the in-hospital mortality rate was 19.4%. The median age of patients who died was 79 (39–99) years, which was significantly higher than that of survivors [71 (19–96) years; *p* < 0.001]. The demographic and clinical characteristics of the patients are presented in Table 1.

Dyspnea was the most common presenting symptom in the overall cohort. Syncope and altered mental status were more frequently observed in patients who died (both *p* < 0.001). Regarding risk factors, immobilization (57.3%), hospitalization within the previous three months (35.9%), and a history of malignancy (23.3%) were most common among patients who died, whereas immobilization (45.1%), recent hospitalization (32.1%), and a history of deep vein thrombosis/pulmonary embolism (DVT/PE) (18.3%) were more frequent among survivors. Immobilization was significantly more prevalent in the mortality group (*p* = 0.040), while a history of DVT/PE was more common in survivors (*p* = 0.013).

Hypertension, atrial fibrillation (AF), and cerebrovascular disease were the most frequent comorbidities in both groups. The prevalence of AF (42.7%) and cerebrovascular disease (28.2%) was significantly higher among patients who died (*p* < 0.001 and *p* = 0.006, respectively).

Echocardiographic evaluation demonstrated that right ventricular dysfunction was significantly more common in patients with in-hospital mortality (62.1%; *p* < 0.001).

Physical examination findings and laboratory parameters at emergency department presentation are summarized in Table 2. Median heart rate and respiratory rate were significantly higher in patients who died, whereas systolic and diastolic blood pressure values were significantly lower (all *p* < 0.001). Arterial lactate levels were significantly higher in patients who died compared with survivors [4.1 (1–15.6) vs. 1.9 (0.5–7.7) mmol/L; *p* < 0.001]. Similarly, troponin I levels were significantly higher in the mortality group [0.32 (0.001–2.1) vs. 0.17 (0.001–1.9) µg/L; *p* = 0.006]. The PESI score was also significantly higher among patients who died [179 (79–275) vs. 119 (27–244); *p* < 0.001].

When arterial lactate levels were categorized as <2, 2–4, and >4 mmol/L, a significant stepwise association between increasing lactate levels and in-hospital mortality was observed (*p* < 0.001). Among patients who died, 79.6% had lactate levels ≥2 mmol/L (2–4 mmol/L: 50.5%; >4 mmol/L: 29.1%), whereas 63.8% of survivors had lactate levels <2 mmol/L.

Among normotensive patients at emergency department presentation (systolic blood pressure ≥100 mmHg; *n* = 211), those with lactate ≥2 mmol/L had a significantly higher in-hospital mortality rate compared with those with lactate <2 mmol/L (35.7% vs. 8.7%; *p* < 0.001) (Table 3).

**Figure 1:**
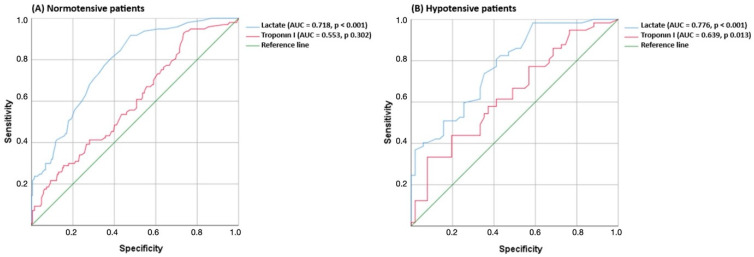
ROC analysis of arterial lactate and troponin levels for predicting in-hospital mortality.

In both normotensive and hypotensive subgroups, receiver operating characteristic analysis demonstrated that arterial lactate had greater discriminative ability for predicting in-hospital mortality than troponin I (normotensive: AUC 0.718 vs. 0.553; hypotensive: AUC 0.776 vs. 0.639) (Figure 1). Troponin I was not statistically significant for predicting mortality in normotensive patients (*p* = 0.302).

**Figure 2:**
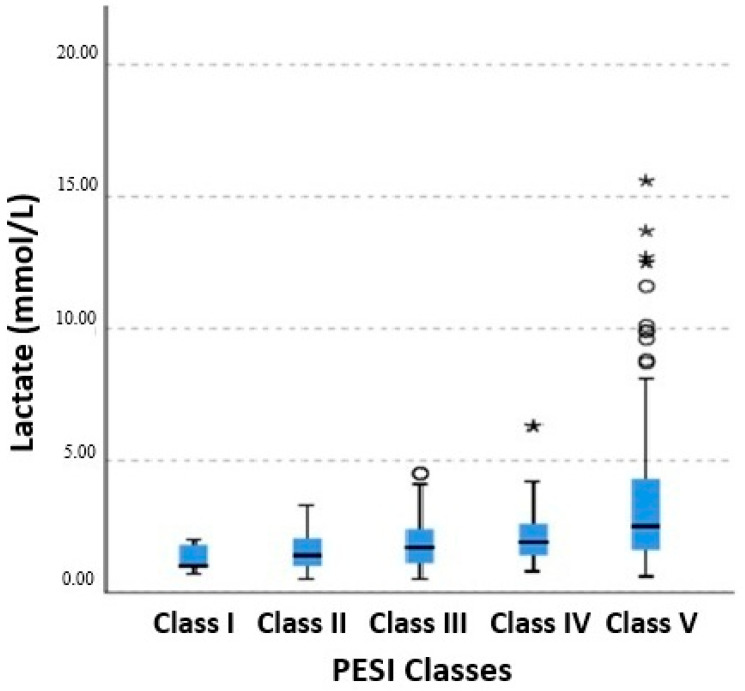
Distribution of arterial lactate levels according to PESI classes. A progressive increase in lactate levels was observed with increasing PESI class. Intergroup comparisons were performed using the Kruskal–Wallis test (*p* < 0.001). Outliers are shown as circles (○), and extreme outliers are shown as asterisks (*).

**Figure 3:**
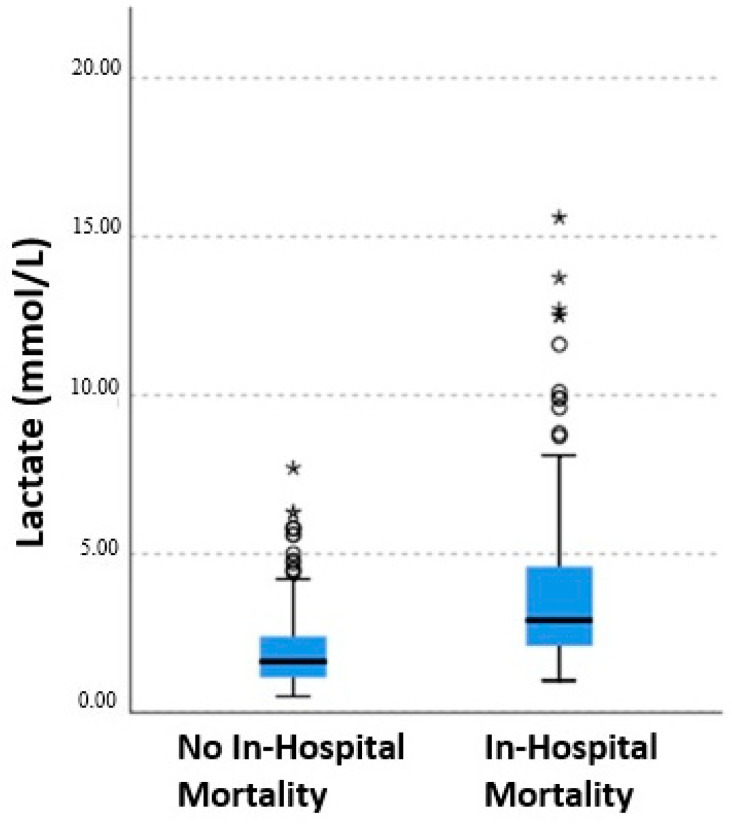
Distribution of arterial lactate levels according to in-hospital mortality status. Outliers are shown as circles (○), and extreme outliers are shown as asterisks (*).

According to the European Society of Cardiology risk classification, among patients who died, 61 (59.2%) were categorized as high risk, 5 (4.9%) as intermediate–high-risk, and 36 (35.0%) as intermediate–low-risk, whereas only 1 patient (1.0%) was classified as low risk. The majority of patients who died were in PESI classes IV and V, with 79.6% (*n* = 82) classified as PESI class V. A progressive increase in in-hospital mortality was observed across PESI classes from low to high risk. Similarly, arterial lactate levels increased progressively from PESI class I to class V (Figure 2). Arterial lactate levels were significantly higher in non-survivors compared with survivors, demonstrating a strong association between elevated lactate and in-hospital mortality (Figure 3).

In multivariable logistic regression analysis performed in normotensive patients, the presence of cerebrovascular disease (OR 3.218, 95% CI 1.184–8.744; *p* = 0.022), arterial lactate level (OR 1.627, 95% CI 1.087–2.436; *p* = 0.018), and PESI score (OR 1.026, 95% CI 1.015–1.037; *p* < 0.001) were identified as independent predictors of in-hospital mortality (Table 4). Each 1 mmol/L increase in arterial lactate was associated with an approximately 63% increase in mortality risk. In contrast, syncope (*p* = 0.801), atrial fibrillation (*p* = 0.072), and troponin level (*p* = 0.120) were not independently associated with mortality.

## 4. Discussion

In the present study, arterial lactate levels measured at emergency department admission in patients diagnosed with acute pulmonary embolism were found to be independently associated with in-hospital mortality. In particular, multivariable analysis in normotensive patients demonstrated that arterial lactate level was independently associated with in-hospital mortality. In acute pulmonary embolism, elevated lactate levels are thought to reflect not only systemic hypotension but also early manifestations of reduced cardiac output due to right ventricular pressure overload, microcirculatory hypoperfusion, and tissue hypoxia [13]. The association between elevated lactate levels and mortality even in normotensive patients suggests the presence of tissue hypoperfusion in the absence of systemic hypotension and may serve as an early indicator of clinical deterioration [14]. Hemodynamic compromise in acute pulmonary embolism is not limited to overt hypotension [3,15]. A subset of normotensive patients may present with a clinical condition characterized by reduced cardiac index and tissue hypoperfusion, referred to as “normotensive shock” [16,17]. In these patients, the presence of blood pressure within normal limits may mask the underlying circulatory failure and lead to delayed recognition of a high-risk state [7,18]. In this context, our findings suggest that lactate may serve as a biochemical marker of normotensive shock, particularly in clinically stable-appearing normotensive patients, and may contribute to the early identification of occult hemodynamic deterioration. In our study, lactate levels ≥2 mmol/L in normotensive patients were associated with an approximately sixfold higher risk of mortality, supporting this pathophysiological mechanism. Importantly, our study extends the existing literature by specifically focusing on normotensive patients and by directly comparing lactate with conventional biomarkers, demonstrating its independent association and incremental prognostic value in a clinically relevant subgroup.

According to the European Society of Cardiology guidelines, risk stratification in intermediate-risk PE is based on clinical scores (PESI and simplified Pulmonary Embolism Severity Index [sPESI]), cardiac biomarkers, and echocardiographic findings [3]. Although PESI and sPESI have high negative predictive value, their specificity for predicting mortality is limited. These scores predominantly incorporate variables based on age and comorbidities and do not directly reflect the acute hemodynamic burden of pulmonary embolism [7,19]. Therefore, they may not be sufficient when used alone to guide decisions regarding advanced therapeutic strategies. Troponin levels, as markers of cardiac injury, are associated with short-term mortality in acute PE; however, when evaluated alone, their specificity and positive predictive value are low [7,20]. Troponin elevation, although a marker of myocardial stress secondary to right ventricular pressure overload, may also be elevated in various clinical conditions, including acute coronary syndromes, chronic kidney disease, heart failure, sepsis, atrial fibrillation, and advanced age [20,21,22]. Therefore, the current literature suggests that the use of troponin alone is not sufficient and that its combination with clinical scores and imaging modalities demonstrating right ventricular dysfunction may improve prognostic accuracy [3,23,24]. In our study, although troponin levels were associated with mortality in univariable analysis, they did not retain independent significance in multivariable analysis performed in normotensive patients. Elevated troponin levels in acute pulmonary embolism are known to be strongly associated with mortality. However, it has been reported that troponin may be insufficient to distinguish between high- and low-risk patients in hemodynamically stable populations [25]. In this context, the lack of independent prognostic significance of troponin in our multivariable model may be explained by several factors. Troponin primarily reflects myocardial injury secondary to right ventricular pressure overload, whereas lactate represents a more global marker of systemic hypoperfusion and impaired tissue perfusion. In addition, troponin elevation may be influenced by a wide range of comorbid conditions and may overlap with variables included in composite clinical scores such as PESI, thereby reducing its incremental contribution in multivariable models. This finding suggests that the independent prognostic value of troponin may be limited in normotensive patients. In the normotensive subgroup, among patients with normal troponin levels, mortality was significantly higher in those with lactate ≥2 mmol/L compared to those with lactate <2 mmol/L (38.1% vs. 5.6%; *p* = 0.002), corresponding to an approximately 6.9-fold increase in mortality risk. Similarly, among patients with elevated troponin levels, mortality remained significantly higher in those with lactate ≥2 mmol/L compared to those with lactate <2 mmol/L (35.0% vs. 11.4%; *p* < 0.001). These findings suggest that lactate may help identify a high-risk subgroup even among normotensive patients who might otherwise be considered low risk based on conventional biomarkers.

Additionally, the identification of cerebrovascular disease as the strongest independent predictor of mortality in the normotensive subgroup highlights the important role of comorbidity burden in determining prognosis. In our study, the markedly higher mortality rate observed in patients with cerebrovascular disease (41.2% vs. 15.3%) suggests that this group represents a more fragile clinical phenotype. The older age of these patients, higher rates of immobilization, increased frequency of altered mental status, and a greater burden of accompanying comorbidities may be associated with reduced physiological reserve and increased susceptibility to acute stress. Notably, previous studies have also demonstrated that mortality is significantly increased in patients with pulmonary embolism accompanied by ischemic stroke, and that this patient group is characterized by a higher burden of comorbidities [26]. Therefore, cerebrovascular disease may be considered not as a direct causal factor but rather as a marker of a more fragile patient profile. However, residual confounding cannot be completely excluded, as variables such as frailty, functional status, and the severity of prior cerebrovascular events were not directly assessed in this study.

Echocardiography is an important tool for the assessment of right ventricular dysfunction in acute PE; however, it has certain limitations. A recent meta-analysis reported substantial heterogeneity among echocardiographic parameters used to define right ventricular dysfunction and the absence of a standardized definition [12]. In addition, the method is largely operator-dependent, and the requirement for experienced personnel constitutes an important factor limiting its routine use [12,27]. In the Registro Informatizado de la Enfermedad TromboEmbólica (RIETE) registry based on real-world data, early transthoracic echocardiography performed within the first 72 h in acute PE was frequently used in clinical practice, and certain echocardiographic findings were associated with poor clinical outcomes. However, it has been reported that the patients selected for echocardiography and the timing of its performance after diagnosis varied considerably, with differences related to both patient selection and timing; notably, echocardiographic evaluation was performed in only 42.8% of patients [28]. Consistent with these findings, the lack of 24/7 availability of echocardiography in many centers and its potential to cause delays at the time of acute presentation may limit its practical use in early risk assessment. Therefore, echocardiographic findings are recommended to be interpreted in combination with clinical scores and biomarkers rather than used alone to guide clinical decision-making [3,12,28]. For these reasons, additional, readily accessible, and objective prognostic markers are needed to identify normotensive PE patients at risk of early clinical deterioration. The higher discriminative performance of lactate compared to troponin in normotensive patients (AUC 0.718 vs. 0.553) may be attributable to non-standardized timing of biomarker measurement, differences in biomarker kinetics, and potential residual confounding. Nevertheless, these findings suggest that lactate may serve as a valuable biomarker providing complementary and clinically meaningful prognostic information, particularly in normotensive patients, rather than representing a superior alternative to troponin.

Recent studies have demonstrated the direct biological association between lactate and the thromboembolic process. Ząbczyk et al. demonstrated that higher lactate levels were associated with a denser, fibrinolysis-resistant thrombus structure [29]. Similarly, Maekawa et al. reported that lactate levels were increased in newly developing, erythrocyte-rich venous thrombi [30]. These findings suggest that lactate elevation in acute PE is not merely a consequence of systemic hemodynamic impairment but may also reflect the biological activity of the thromboembolic burden. Particularly in normotensive patients, it is understood that lactate elevation associated with thrombus-related metabolic activity may occur before the development of hemodynamic instability and may provide early prognostic information.

It is observed that the prognostic cutoff values for lactate in acute pulmonary embolism reported in the literature are heterogeneous. Ebner et al. reported a venous lactate threshold of ≥3.3 mmol/L, while other studies and systematic evidence have shown that elevated lactate levels, including arterial lactate values around ≥2.0 mmol/L, are associated with short-term adverse outcomes in acute pulmonary embolism [14,31,32]. These differences may be explained by variations in sampling methods, timing of measurement, and the risk profiles of the study populations. In our study, the prognostic significance of a threshold of ≥2 mmol/L in normotensive patients supports the notion that lactate may contribute to risk discrimination even at relatively low threshold levels in the early phase.

When lactate levels were evaluated categorically, it was observed that cases with mortality were concentrated in higher lactate ranges, and mortality increased progressively as lactate levels increased. Moreover, the parallel increase in lactate levels with increasing PESI classes indicates that lactate is biologically concordant with clinical risk scores and reflects the underlying pathophysiological burden associated with increasing clinical risk. When lactate levels were analyzed according to ESC risk categories, the median value was 2.75 (0.80–15.60) mmol/L in the high-risk group, 1.75 (0.50–5.80) mmol/L in the intermediate–high-risk group, 1.80 (0.50–7.90) mmol/L in the intermediate–low-risk group, and 1.00 (0.50–2.80) mmol/L in the low-risk group. There was a statistically significant difference among low-, intermediate-, and high-risk groups overall (*p* < 0.001); however, no significant difference was observed between the intermediate–high and intermediate–low-risk groups (*p* = 0.454). However, within the intermediate-risk group, lactate levels were significantly higher in patients who died compared to survivors (2.4 vs. 1.6 mmol/L, *p* < 0.001), suggesting that lactate may provide additional prognostic value within the intermediate-risk category.

In multivariable analysis performed in normotensive patients, the persistence of an independent association between lactate level and mortality, in contrast to the loss of statistical significance of troponin, may be explained by the differences in the pathophysiological processes reflected by these biomarkers in acute pulmonary embolism. Troponin elevation primarily reflects right ventricular myocardial injury and acute pressure overload, whereas lactate level is a more global indicator of systemic perfusion impairment and tissue hypoperfusion. In normotensive patients, microcirculatory perfusion abnormalities may occur even before overt right ventricular dysfunction or myocardial necrosis develops, and this condition may be detected by elevated lactate levels. Therefore, lactate may have retained its independent prognostic value in multivariable models as a biomarker reflecting reduced hemodynamic reserve and early circulatory failure beyond right ventricular overload. This finding supports the importance of a multimarker approach in acute pulmonary embolism that integrates clinical scores, cardiac biomarkers, and markers of perfusion rather than relying on a single biomarker for risk assessment.

In light of these findings, lactate measurement at emergency department presentation may represent a rapid, inexpensive, and readily available tool for risk stratification in patients with pulmonary embolism. In particular, normotensive patients with lactate ≥2 mmol/L, despite appearing clinically stable, may have an increased risk of mortality and should be carefully considered for closer monitoring, early intensive care evaluation, and aggressive therapeutic strategies.

### Limitations

The principal limitations of this study are its retrospective and single-center design, which may limit the generalizability of the findings to broader patient populations. The inclusion of patients with acute pulmonary embolism who underwent arterial blood gas analysis at presentation may have resulted in the selection of a higher-risk cohort and introduced potential selection bias. Arterial blood gas analysis was not performed routinely in all patients but was obtained based on clinical judgment, particularly in those with more severe clinical presentation; this may have further contributed to selection bias. Therefore, the reported mortality rates were higher compared with those observed in large registry-based studies. Nevertheless, the study provides clinically relevant insights by reflecting real-world data and offering a detailed analysis of the normotensive patient subgroup.

## 5. Conclusions

These findings suggest that lactate elevation in acute pulmonary embolism is not merely a consequence of hemodynamic deterioration but may also reflect the biological burden of the thromboembolic process and provide prognostic information from the early stages of the disease. Arterial lactate level, particularly in normotensive patients with pulmonary embolism, may reflect occult tissue hypoperfusion and may offer incremental prognostic value beyond existing risk stratification systems, thereby serving as a practical adjunct in early clinical risk assessment. These findings provide clinically relevant evidence that arterial lactate is associated with mortality and may offer incremental prognostic information beyond established risk stratification systems, particularly in normotensive patients, thereby addressing an important gap in the current literature.

## Figures and Tables

**Table 1:** Demographic and Clinical Characteristics of the Patients According to In-Hospital Mortality Status.

Variable	In-Hospital Mortality (*n* = 103)	No In-Hospital Mortality (*n* = 224)	*p*-Value
Age, median (min–max)	79 (39–99)	71 (19–96)	**<0.001**
Sex (female/male)	67/36	150/74	0.733
Symptoms, *n* (%)			
Dyspnea	95 (92.2%)	201 (89.7%)	0.473
Chest pain	14 (13.6%)	55 (24.6%)	**0.024**
Syncope	50 (48.5%)	58 (25.9%)	**<0.001**
Change in consciousness	63 (61.2%)	43 (19.2%)	**<0.001**
Leg swelling	19 (18.4%)	47 (21.0%)	0.596
Hemoptysis	6 (5.8%)	8 (3.6%)	0.350
Risk Factors, *n* (%)			
Immobility	59 (57.3%)	101 (45.1%)	**0.040**
Hospitalization (last 3 months)	37 (35.9%)	72 (32.1%)	0.501
History of DVT/PE	8 (7.8%)	41 (18.3%)	**0.013**
Malignancy	24 (23.3%)	38 (17.0%)	0.175
Recent surgery (last 3 months)	19 (18.4%)	33 (14.7%)	0.394
Recent trauma (last 1 month)	4 (3.9%)	8 (3.6%)	0.889
Long-term travel	5 (4.9%)	13 (5.8%)	0.727
Comorbidities, *n* (%)			
Hypertension	64 (62.1%)	124 (55.4%)	0.249
Cerebrovascular disease	29 (28.2%)	34 (15.2%)	**0.006**
Coronary artery disease (CAD)	17 (16.5%)	28 (12.5%)	0.329
Alzheimer’s disease	16 (15.5%)	31 (13.8%)	0.685
Diabetes mellitus (DM)	10 (9.7%)	25 (11.2%)	0.693
Congestive heart failure	17 (16.5%)	24 (10.7%)	0.142
Atrial fibrillation (AF)	44 (42.7%)	42 (18.8%)	**<0.001**
Asthma	3 (2.9%)	17 (7.6%)	0.101
Chronic obstructive pulmonary disease (COPD)	14 (13.6%)	23 (10.3%)	0.378

Bold values indicate statistical significance (*p* < 0.05).

**Table 2:** Clinical and Laboratory Characteristics of the Patients According to In-Hospital Mortality Status.

	In-Hospital Mortality (*n* = 103) Median (Min–Max)	No In-Hospital Mortality (*n* = 224) Median (Min–Max)	*p*-Value
Pulse	108 (50–160)	96 (55–160)	**<0.001**
Systolic Blood Pressure (SBP)	99 (70–150)	117 (70–190)	**<0.001**
Diastolic Blood Pressure (DBP)	64 (40–100)	73 (37–120)	**<0.001**
Respiratory Rate (RR)	27 (10–53)	23 (15–40)	**<0.001**
Arterial blood gas			
pH	7.40 (6.96–7.52)	7.42 (7.13–7.6)	**<0.001**
PaCO_2_ (mmHg)	41.8 (19–103)	37 (19–84)	**0.014**
PaO_2_ (mmHg)	65.7 (44–95)	64.4 (47–94)	0.594
SaO_2_ (%)	89 (39–99)	89 (50–99)	0.668
Lactate (mmol/L)	4.1 (1–15.6)	1.9 (0.5–7.7)	**<0.001**
Haemogram			
WBC (10^3^/µL)	13.1 (3.1–33)	10.4 (2.3–31.1)	**0.001**
HGB (g/dL)	12 (7.7–17.1)	12.4 (7–18.3)	0.146
PLT (10^3^/µL)	229 (33–786)	226 (55–627)	0.789
Other			
Creatinine (mg/dL)	1.2 (0.4–3.5)	0.9 (0.3–8)	**<0.001**
CRP (mg/L)	48.6 (0–284)	46.9 (0–348)	0.691
D-dimer (µg FEU/L)	6541 (108–72454)	6345 (156–51800)	0.146
Troponin I (µg/L)	0.32 (0.001–2.1)	0.17 (0.001–1.9)	**0.006**
Echocardiographic findings			
PASP (mmHg)	35 (10–90)	36 (10–85)	0.990
LVEF	57 (20–65)	58 (30–68)	0.128
D-shaped septum, *n* (%)	37 (35.9%)	69 (31.8%)	0.330
Right ventricular overload, *n* (%)	64 (62.1%)	88 (40.4%)	**<0.001**
PESI score	179 (79–275)	119 (27–244)	**<0.001**

Pulse: beats per minute (bpm); SBP and DBP: mmHg; Respiratory rate (RR): breaths per minute; SaO_2_: percentage (%); WBC: White Blood Cell; HGB: Hemoglobin concentration; PLT (10^3^/µL): Platelet count; CRP: C-reactive protein; PaCO_2_: partial pressure of arterial carbon dioxide; PaO_2_: partial pressure of arterial oxygen; PASP: pulmonary artery systolic pressure; LVEF: left ventricular ejection fraction; FEU: fibrinogen equivalent units; PESI: Pulmonary Embolism Severity Index. Bold values indicate statistical significance (*p* < 0.05).

**Table 3:** Prognostic Value of Lactate in Normotensive Patients (SBP ≥ 100 mmHg).

	Number of Patients (*n* = 211)	In-Hospital Mortality, *n* (%)	OR (95% CI)	*p*-Value
Lactate, *n* (%)				
<2 mmol/L	127	11 (8.7%)	Reference	
≥2 mmol/L	84	30 (35.7%)	5.8 (2.7–12.5)	**<0.001**

CI: confidence interval, OR: Odds ratio. The reference group was <2 mmol/L. Bold values indicate statistical significance (*p* < 0.05).

**Table 4:** Multivariable Logistic Regression Analysis for In-Hospital Mortality in Normotensive Patients.

Variable	OR	95% CI	*p*-Value
Syncope	0.881	0.328–2.364	0.801
Atrial fibrillation	2.209	0.931–5.241	0.072
Cerebrovascular disease	3.218	1.184–8.744	**0.022**
Troponin I (continuous)	2.778	0.766–10.071	0.120
Lactate (per 1 mmol/L increase)	1.627	1.087–2.436	**0.018**
PESI score (per 1-point increase)	1.026	1.015–1.037	**<0.001**

CI: confidence interval, OR: Odds ratio. Bold values indicate statistical significance (*p* < 0.05).

## Data Availability

The datasets used and analyzed during the current study are available from the corresponding author on reasonable request.
